# Examining the link between tooth loss and multiple health outcomes: a comprehensive synthesis of evidence from meta-analyses

**DOI:** 10.3389/fpubh.2026.1857651

**Published:** 2026-08-12

**Authors:** Siping Song, Wanqing Xie, Shuqi Huang, Jing He, Lixian Chen, Fan Liu

**Affiliations:** 1 Department of Oral and Maxillofacial Surgery, State Key Laboratory of Oral Diseases and National Clinical Research Center for Oral Diseases, West China Hospital of Stomatology, Sichuan University, Chengdu, Sichuan, China; 2 Post-anesthetic care unit, State Key Laboratory of Oral Diseases and National Clinical Research Center for Oral Diseases, West China Hospital of Stomatology, Sichuan University, Chengdu, Sichuan, China; 3 Nursing Department, State Key Laboratory of Oral Diseases and National Clinical Research Center for Oral Diseases, West China Hospital of Stomatology, Sichuan University, Chengdu, Sichuan, China; 4 Department of Oral Implantology, State Key Laboratory of Oral Diseases and National Clinical Research Center for Oral Diseases, West China Hospital of Stomatology, Sichuan University, Chengdu, Sichuan, China

**Keywords:** general health, meta-analyses, oral health, review, tooth loss

## Abstract

**Objectives:**

Evidence linking tooth loss to various systemic health consequences is summarized and evaluated in this umbrella review. Evidence gaps are identified, and recommendations for the development of public oral health measures are provided.

**Methods:**

This prospective registered umbrella review was conducted in accordance with the PRISMA guidelines. To identify meta-analyses of observational studies that have explored the link between tooth loss and systemic health outcomes, systematic literature searches were conducted in PubMed, the Web of Science, MEDLINE, and the Cochrane Library from the inception of the databases through September 2025. Methodological quality and evidence strength were evaluated using AMSTAR-2, GRADE, and credibility assessments.

**Results:**

An examination of 27 meta-analyses containing 40 distinct estimates revealed that tooth loss was linked to increased risks of cardiovascular diseases, several types of cancer, neurodegenerative diseases, metabolic disorders, mental disorders, and respiratory diseases. Dose–response analyses revealed incremental risks with increasing numbers of teeth lost for several outcomes, including cardiovascular diseases, all-cause mortality, esophageal cancer, lung cancer, colorectal cancer, cognitive decline, and dementia. However, the comprehensive evidence quality was predominantly rated as low or very low, with significant heterogeneity and potential biases noted.

**Conclusion:**

This umbrella review synthesizes evidence demonstrating that tooth loss is consistently linked to increased risks of multiple detrimental health consequences, underscoring its importance in systemic health rather than merely being a local dental issue. Oral health and holistic health management, especially among older adults people, should be considered to help reduce the risk of various systemic illnesses.

**Systematic review registration:**

https://www.crd.york.ac.uk/PROSPERO/view/CRD420251149566

## Introduction

Aging-related diseases are becoming increasingly prevalent and have significant socioeconomic impacts because of the increased life expectancy worldwide (1, 2). Oral diseases are among the most critical global health issues because they chronically and progressively affect the teeth and oral cavity and have significant effects on the holistic health of individuals worldwide (3, 4). According to the Global Burden of Diseases report, the worldwide prevalence of severe tooth loss (defined as having ≤ 9 remaining teeth) is 2.4%, affecting approximately 2% of the global population (5). Severe tooth loss ranks 36th among the most prevalent chronic diseases associated with reduced life expectancy (5). Moreover, the World Health Organization (WHO) has repeatedly noted the importance of including preventative oral health measures in universal health coverage programs.

As critical chronic and progressive oral health issues, oral diseases significantly impact individuals’ overall health (3, 4). Substantial evidence has shown that poor oral health is strongly associated with impaired masticatory function, suboptimal nutritional status, unhealthy dietary changes, diminished self-esteem, reduced quality of life, and adverse systemic health outcomes (6–9). Chronic periodontitis, the sixth most prevalent noncommunicable disease worldwide, is a chronic inflammatory condition resulting from oral microbial dysbiosis that leads to the destruction of the periodontal ligament and alveolar bone, ultimately leading to tooth loss (10). Severe periodontitis is a primary cause of tooth loss in adults, and this loss, representing the endpoint outcome of cumulative oral inflammation and a lifetime of dental disease (11, 12), has recently been recognized as having a significant bidirectional association with systemic health (2). For example, impaired masticatory function caused by tooth loss is strongly associated with malnutrition. Moreover, changes in the diet of affected individuals severely increase the risk of high blood pressure, diabetes, cardiovascular disease and other diseases (13, 14). Furthermore, facial changes associated with missing teeth negatively affect social interactions, which leads to decreased self-esteem and other psychological problems. In particular, tooth loss is related to increased risks of cognitive impairment, cancer and premature mortality, which can negatively affect quality of life (13, 15).

In recent decades, many meta-analyses have established associations between tooth loss and various health outcomes. However, deficiencies in research design, the use of different measurement methodologies, incompatible research findings and diverse explanations of exposure pose challenges in reaching conclusive determinations. A comprehensive review may increase our understanding of these associations and provide systematic insights to improve the quality (16), strength and validity of the available comprehensive evidence on the health outcomes associated with tooth loss.

To bridge this gap, we conducted a review to assess the strength, credibility, and potential bidirectionality of the associations between tooth loss and systemic diseases while systematically analyzing the specific health outcomes involved. Furthermore, we investigated whether future studies are likely to change the conclusions derived from existing meta-analyses.

## Methods

### Protocol and registration

We performed a detailed review to assess the quality and possible biases of available studies in which the relationships between tooth loss and various health outcomes were investigated. This approach allows for the systematic capture and evaluation of existing evidence across multiple health domains. We performed this umbrella review in compliance with the Preferred Reporting Items for Systematic reviews and Meta-Analyses (PRISMA) statement for systematic reviews and meta-analyses, and the protocol was prospectively registered in PROSPERO (CRD420251149566) (17).

### Literature search

An integrated search was conducted in PubMed, the Web of Science, MEDLINE, and the Cochrane Library from the inception of the databases until September 2025 to obtain systematic reviews and meta-analyses. In the literature search, we used medical subject heading (MeSH) terms, keywords, and different textual terms linked to tooth loss (“tooth loss” OR “edentulism”) AND (“systematic review” OR “meta-analysis”) (Supplementary Table S1). Furthermore, we searched for gray literature using the platform at http://www.opengrey.eu. The literature screening was performed by two independent reviewers (S. P. and X. W.) who separately reviewed the titles and abstracts of the studies and identified meta-analyses that met the eligibility criteria by reading the full text. They subsequently reviewed the full texts of the remaining studies together to ensure that they met the final criteria for inclusion. All discrepancies were adjudicated by a third reviewer (F. L.). We manually examined the reference sections of each included article and reviewed the included meta-analyses and reviews. This step helped us identify all the studies that were relevant to our research.

### Eligibility criteria

We selected systematic reviews with meta-analyses that examined how tooth loss (either exposure or endpoint) is related to various health issues; we also considered cohort, case–control, and cross-sectional studies. For several studies that explored the same links between tooth loss and health outcomes and were published more than two years apart, we included the most recent study in our analysis because the more recent study typically had the largest participant group. When several relevant studies were released within a two-year period, we chose the meta-analysis with the largest sample and the most prospective cohort investigations. If these studies had the same number of participants in the prospective cohorts, we included the study with the higher AMSTAR rating. Furthermore, if the most recent study did not include a dose–response analysis but another study did include such analysis, we included both studies (18).

We did not set any limits on the language of the published papers. Our exclusion criteria were as follows: (i) systematic reviews that had no meta-analyses and in which quality was not measured; (ii) reviews that did not provide meta-analytic figures and heterogeneity details; and (iii) umbrella reviews that collated results from other systematic reviews. All health outcomes were prespecified on the basis of a preliminary review of existing systematic reviews and remained unchanged throughout the study selection process.

### Data extraction

We collected important details from the published meta-analyses included in our review, including the name of the first author, publication year, country, health outcomes, whether tooth loss served as the exposure or the outcome being measured, participant information, quality/bias assessment data, follow-up period, number of primary studies, study design, and specific statistical measures applied in the meta-analysis. Moreover, we gathered specific information about the effect models, the degree of heterogeneity, and the assessment of publication bias, which involved the use of funnel plots as well as Egger’s and Begg’s tests.

Evaluating the credibility of the pooled analyses on the basis of evidence classification criteria: We classified the evidence level into four categories in line with preestablished methodological standards, including the *p* value derived from the random-effects meta-analysis, the *p* value derived from Egger’s regression test for asymmetry (used to assess small study effects), the p value derived from the excess significance assessment of the largest study in each meta-analysis, and the total number of cases included in the analyses. Additionally, outcomes were categorized into the following evidence classes: class I (convincing evidence), class II (highly suggestive evidence), class III (suggestive evidence), class IV (weak evidence), and NS (nonsignificant) (16, 19). Supplementary Table S2 shows the comprehensive criteria for classifying the evidence.

Quality assessment of each pooled analysis using GRADE: The Grading of Recommendations, Assessment, Development and Evaluation (GRADE) system was used to grade the evidence of the included outcomes (20). We considered factors such as bias risk, inconsistency, indirectness, imprecision, and publication bias to judge the quality of the evidence, which was divided into four levels: high, moderate, low, and very low.

Quality assessment of individual meta-analysis studies using the AMSTAR-2: The Assessment of Multiple Systematic Reviews (AMSTAR) 2 checklist consists of 16 items that address the entire process of selection, registration, study design, literature search, study selection, data extraction, risk of bias assessment, statistical analysis, and discussion of heterogeneity and publication bias (21). Each item is rated “yes,” “partially yes,” or “no.” We then assigned an overall methodological quality grade of “high,” “moderate,” “low,” or “critically low” to each study.

One author extracted the relevant data, while a second author verified all the details independently. For quality and evidence grading, duplicate assessments were performed independently by two reviewers across the AMSTAR-2, GRADE, and predefined credibility classification frameworks. Any inconsistencies were resolved via consultation with the senior third reviewer.

### Data analysis

This review provides a descriptive summary of the relationships between tooth loss and multiple health outcomes. We extracted pooled effect sizes along with 95% confidence intervals (CIs) from the included meta-analyses, which were computed via random effects or fixed effects frameworks. We used the I-squared (*I*^2^) value to measure the degree of heterogeneity across studies. If sufficient data were provided, we assessed publication bias using funnel plots, Egger’s test, and Begg’s test. We did not reanalyze the other data or primary studies included in the meta-analyses. We used MATLAB software to generate forest plots, which visually present the pooled effect estimates and their 95% CIs, to illustrate the associations between tooth loss and various systemic health outcomes across studies.

## Results

### Description

**Figure 1:**
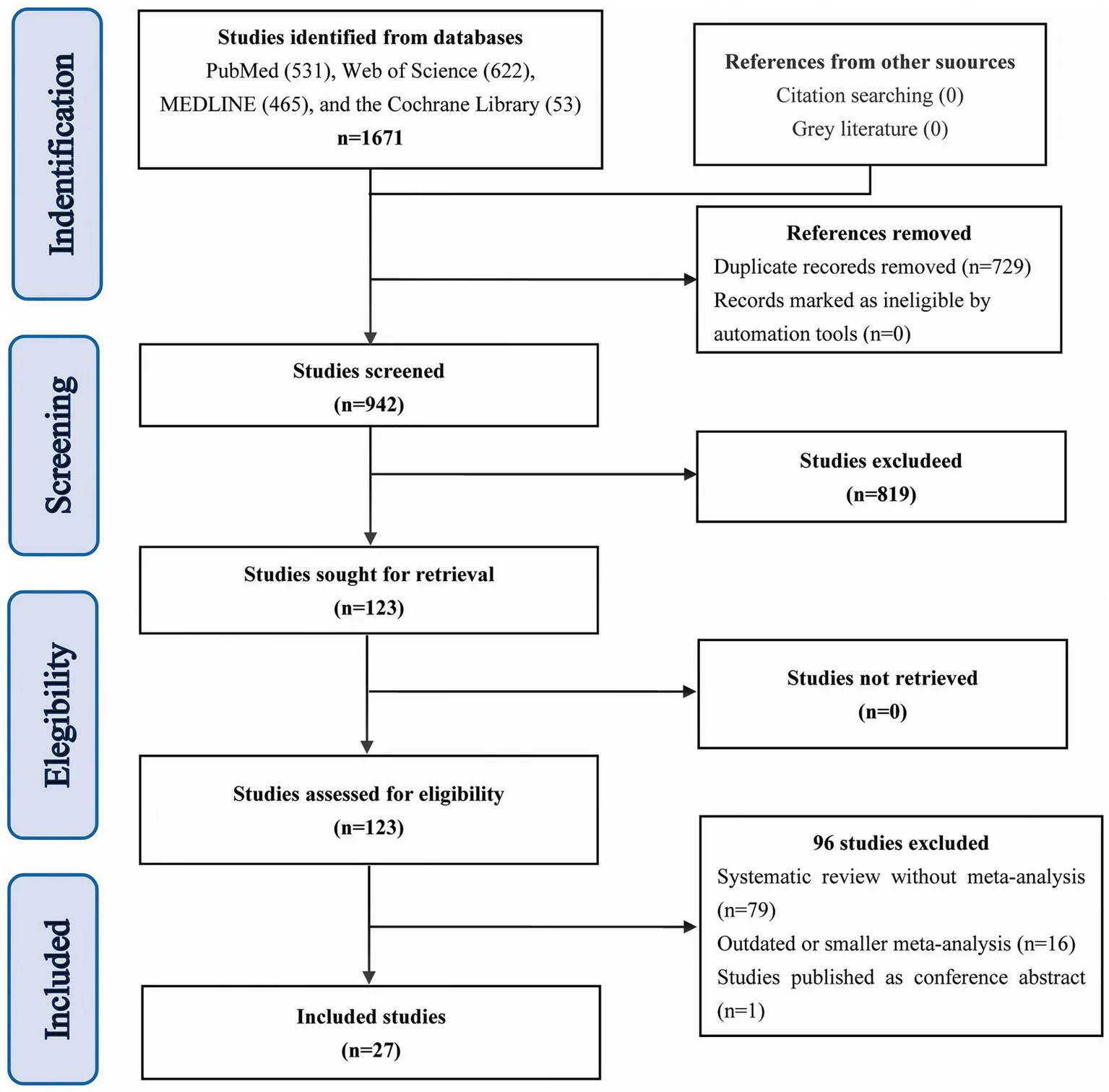
Preferred reporting items for systematic reviews and meta-analyses (PRISMA) flowchart.

Through the initial systematic literature search, we identified a total of 1,671 records. A flow chart outlining the entire literature screening and selection process is shown in Figure 1. After duplicates and ineligible articles were removed, 27 systematic reviews with meta-analyses met our preset inclusion requirements (Table 1). The outcomes from the included reviews were based on 3 to 44 original studies, with 40 separate endpoints identified in these observational meta-analyses. The majority of these meta-analyses focused on the link between tooth loss and cardiovascular diseases (*n* = 14); other analyses focused on the link between tooth loss and neurodegenerative diseases (*n* = 10), cancer (*n* = 8), metabolic disorders (*n* = 4), mental disorders (*n* = 3), and respiratory diseases (*n* = 1), as detailed in Supplementary Table S3.

**Table 1:** Descriptive data of selected systematic reviews and meta-analyses of tooth loss and health outcomes.

Systemic condition	Author (year of publication)	Country	Systemic condition/marker	Tooth loss as an exposure (E) or outcome (O)	Measurement of tooth loss	Participants, n	Quality/bias assessment	Follow-up period	Number of studies in each meta-analysis	Number of cohort/case control /cross-sectional studies	Meta-analysis evaluation metric	Effects model	Publication bias (Funnel plots, Egger’s or Begg’s tests)
Cardiovascular diseases	Beukers et al. (2021) (13)	Netherland	CVD events, All-cause mortality	E	0 teeth remaining vs. 1–32 teeth (ref.)	4,697,021	ROBINS-E	1 to 57 years	44	44/0/0	HR; RR	RE	Performed, not reported
	Cheng et al. (2018) (23) Dose–Response	China	CVD, stroke	E	per 2 teeth loss	879,084	NOS	NA	17	17/0/0	RR	RE	NA
	Humphrey et al. (2008) (25)	USA	CHD	E	0–10 teeth missing	112,273	Third USPSTF	5 to 21 years	7	7/0/0	RR	RE	NA
	Lafon et al. (2014) (30)	France	Stroke	E	Increased tooth loss	1,137 to 51,529	Evaluation grid	12 to 57 years	9	9/0/0	HR; RR	RE	NA
	Xu et al. (2022) (28)	China	Hypertension	O/E	Severe tooth loss as an exposure; hypertension as an exposure	1,224,821	NOS; AHRQ	NA	28	5/1/22	OR	FE; RE	Performed, not reported
	Yang et al. (2018) (29)	China	Peripheral artery disease	O	Number of teeth missing	4,307	NOS	NA	7	1/4/2	RR; WMD	RE	NA
	Dai (2015) (24)	China	Stroke	O	Number of teeth present	1,191	MORE	NA	NA	NA	SD	FE; RE	NA
	Aminoshariae et al. (2024) (22)	USA	CVD mortality	E	Edentulous or less than 10 teeth present	90,305	NOS; AHRQ	3.1 to 49 years	12	12/0/0	HR	RE	Begg’s test, Egger’ test; no bias
	Peng et al. (2019) (26) Dose–Response	China	All-cause mortality	E	each 10-, 20-, 32-tooth loss	306,807	NOS	3.7 to 57 years	18	18/0/0	RR	RE	NA
Cancer	Mahuli et al. (2023) (34)	India	Head and neck cancer	E	lost>5,>16, >20, or only 5 teeth present	20,513	NOS	NA	27	0/27/0	OR	RE	Funnel plots; no bias
	Chen et al. (2015) (31)	China	Esophageal cancer	E	Any lost, 16–32 lost, 24–32 lost, Edentulous	107,028	NOS; AHRQ	NA	10	0/10/0	OR	FE; RE	*p* = 0.974
	Chen et al. (2016) (32) Dose–Response	China	Esophageal cancer	E	Per 1 tooth loss	88,274	NA	NA	10	3/5/2	RR	FE	p>0.05
	Yin et al. (2016) (36)	China	Gastric cancer	E	More tooth loss	245,752,848	NOS	1 to 21 years	9	5/4/0	RR	RE	Begg’s test p>0.05
	Yoon et al. (2022) (37)	USA	Lung cancer	E	Lost>10 teeth; per 5 teeth lost	252,178	NA	mean of 7.6 years	8	7/1/0	HR	FE	Egger’s test, *p* > 0.05
	Xu (2022) (28)	China	Pancreatic cancer	E	More tooth loss	1,352,256	NOS	4 to 41 years	17	0/17/0	HR	RE	Begger’s test, *p* = 0.537; Egger’s test, *p* = 0.002; minor bias
	Ma et al. (2018) (33) Dose–response	China	Colorectal cancer	E	Per 2 teeth loss	160,182	NOS	NA	6	4/2/0	RR	FE	Begger ‘s test, *p* = 0.621; Egger’s test, *p* = 0.239; no bias
Neurodegenerative diseases	Qi et al. (2021) (41) Dose–response	China	Cognitive decline, dementia	E	Per additional tooth lost	38,763	NOS; GRADE	Mean values of 5.3 years and 11.9 years for patients with cognitive impairment and dementia, respectively	14	14/0/0	RR	FE; RE	NA
	Li et al. (2023) (40)	China	Cognitive decline, dementia, Alzheimer’s disease	E	Greater tooth loss, remaining teeth <10, edentulism	356,297	NOS	8.6 years (ranging from 2 to 20 years)	8	8/0/0	RR	FE; RE	Begger’s test, *p* = 0.39; Egger’s test, *p* = 0.11; no bias
	Fang et al. (2018) (39)	China	Dementia	E	the number of missing and present teeth	561,846	mDBs	2.4 to 37 years	21	9/3/9	OR	FE; RE	*p* = 0.07
	Dioguardi et al. (2020) (38)	Italy	Alzheimer’s disease	O	Edentulism	13,472	NOS	NA	6	0/6/0	OR	FE; RE	NA
Metabolic disorders	Ahmadinia et al. (2022) (42)	Iran	Diabetes mellitus	O	Risk of tooth loss	677,532	NOS	NA	22	6/3/13	OR	RE	No bias
	Souza et al. (2019) (44)	Australia	Metabolic syndrome	O/E	Number of teeth present	26,750	NOS; GRADE	2 to 33 years	12	2/0/10	OR; SMD	RE	NA
	Nascimento et al. (2016) (43)	Brazil	Obesity	O/E	Tooth loss as an exposure, risk of tooth loss as an outcome	7,994	CAC JBI	NA	16	16/0/0	OR	RE	No bias
Mental disorders	Sun et al. (2021) (47)	China	Schizophrenia	O	Number of missing teeth	712	NOS; GRADE	NA	3	0/3/0	SMD	FE	NA
	Cademartori et al. (2018) (45)	Brazil	Depression	O	More tooth loss	1,553 to 5,419,019	CAC JBI	NA	16	1/0/15	OR	RE	NA
	Kisely et al. (2016) (46)	Australia	Psychological disorders (depression and anxiety)	O	More tooth loss	334,503	NOS	NA	26	0/0/26	MD; SMD; OR	FE	*p* = 0.507
Respiratory diseases	Shi et al. (2018) (48)	China	Chronic obstructive pulmonary disease	O	Number of remaining teeth	23,960	NOS	NA	14	0/14/0	WMD	RE	Bgger’s test, *p* = 0.979, no bias

AHRQ = Agency for Healthcare Research and Quality; CAC JBI = Critical Appraisal Checklist recommended by the Joanna Briggs Institute; Coronary heart disease = CHD; Cardiovascular diseases = CVD; FE = Fixed effect; HR = Hazard ratio; MD = Mean difference; mDBs = modified Downs and Black Scale; MORE = Methodological Evaluation of Observational Research; NA = Not available; NOS = Newcastle–Ottawa Quality Scale; OR = Odds ratio; RE = Random effects; ROBINS-E Tool = Risk of Bias in Nonrandomized Studies of Exposures; RR = Risk ratio; SMD = Standardized mean difference; USPSTF = US Preventive Services Task Force; WMD = Weighted mean difference.

We observed moderate heterogeneity (*I*^2^ = 50–74.9%) in 6 (15%) and high heterogeneity (*I*^2^ ≥ 75%) in 14 (35%) of the 40 combined analyses, as detailed in Supplementary Table S3. After the application of credible grading criteria and the GRADE framework for evidence judgment, most of the 40 pooled results were categorized as “low” or “very low” quality and fell into evidence classes III, IV, or NS. Five cardiovascular outcomes and 1 neurodegenerative outcome were classified as class II. Among all the evaluated outcomes, only four outcomes (10%) associated with stroke, colorectal cancer, cognitive decline and dementia were rated as “moderate” quality (Supplementary Table S4); neither class I nor high-grade evidence was detected in the review.

### Synthesis of evidence linking tooth loss to cardiovascular diseases

**Figure 2:**
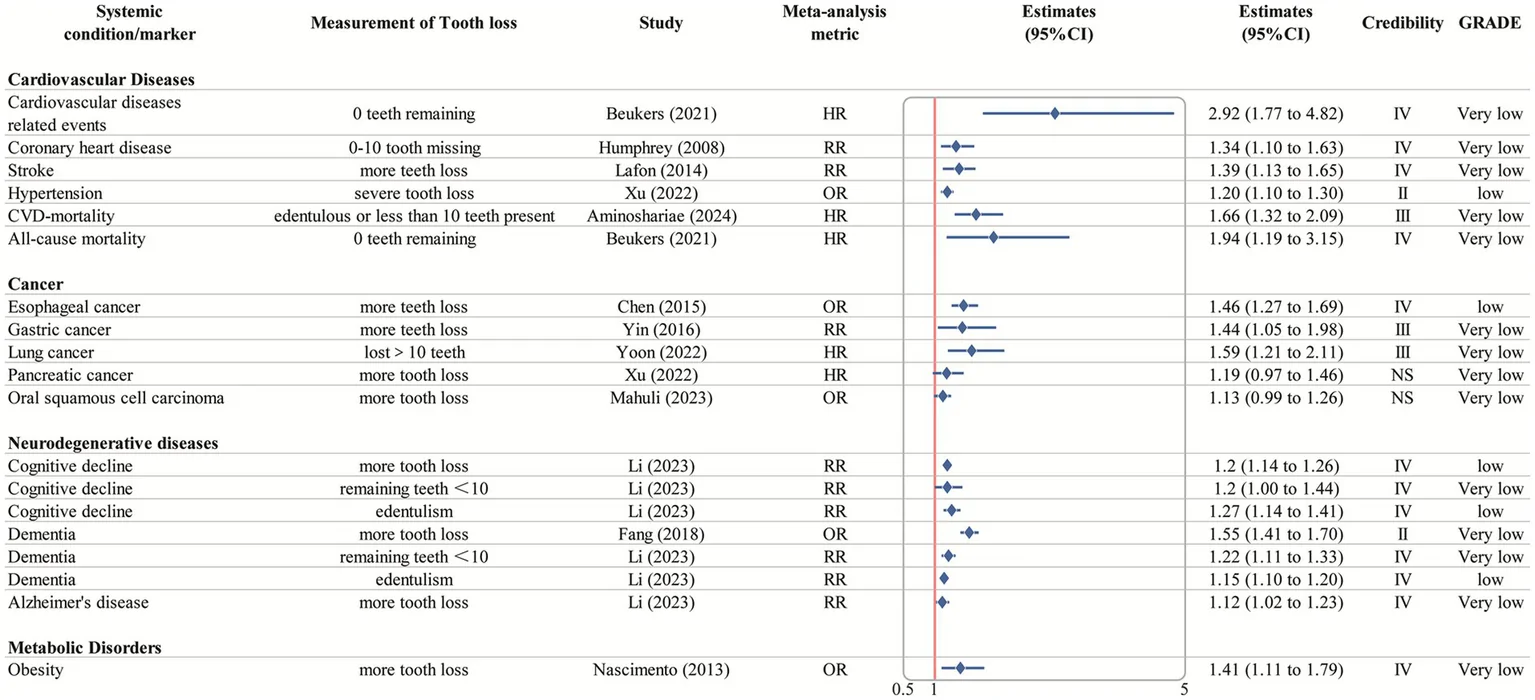
Forest plot of non-dose–response relations between exposure to tooth loss and risk of adverse health outcomes with credibility and GRADE quality assessments. The estimates are expressed in terms of equivalent hazard ratio, odds ratios, and risk ratios. GRADE, grading of recommendations, assessment, development and evaluation; HR, hazard ratio; OR, odds ratio; RR, risk ratio; 95% Cl, 95% confidence intervals.

**Figure 3:**
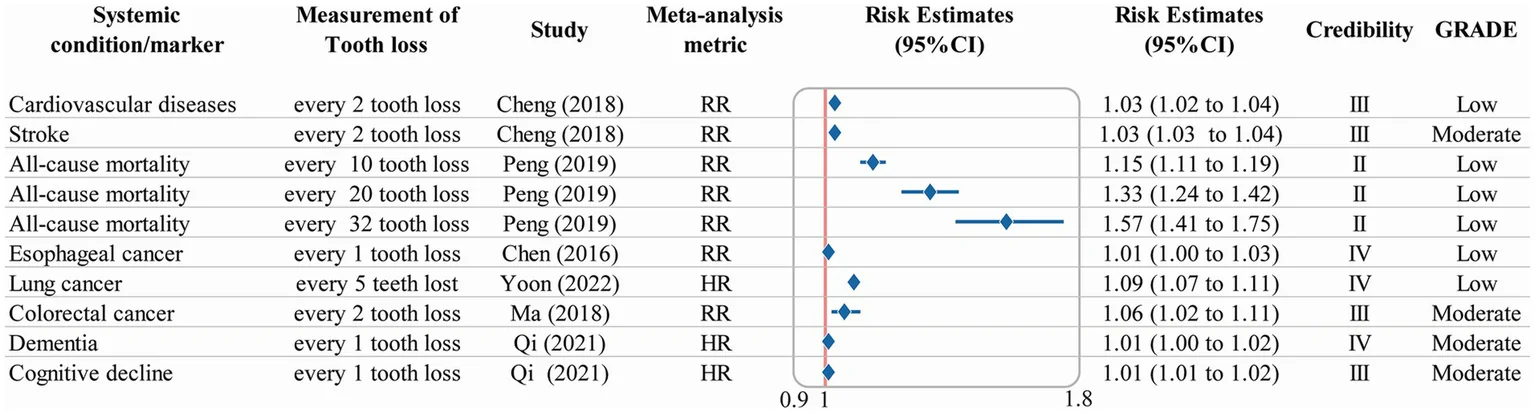
Forest plot of dose–response relations between exposure to tooth loss and risk of adverse health outcomes with credibility and GRADE quality assessments. The estimates are expressed in terms of equivalent hazard ratio, and risk ratios. GRADE, grading of recommendations, assessment, development and evaluation; HR, hazard ratio; RR, risk ratio; 95% Cl, 95% confidence intervals.

**Figure 4:**
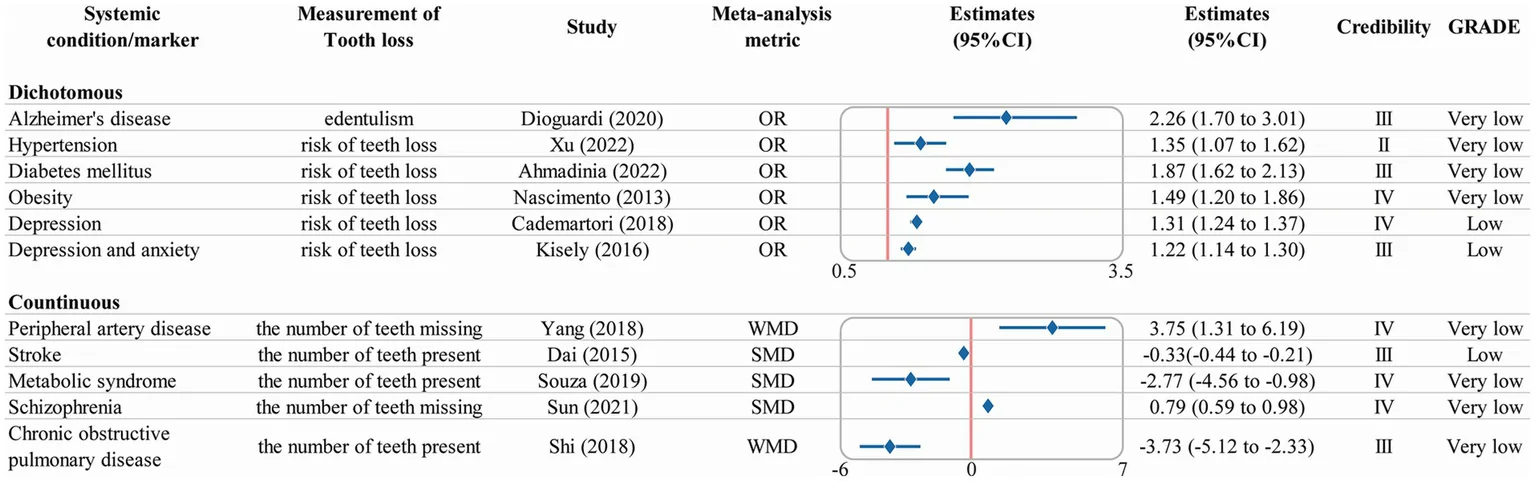
Forest plot of non-dose–response relations between tooth loss as outcome and adverse health with credibility and GRADE quality assessments. The estimates are expressed in terms of equivalent hazard ratio, odds ratios, risk ratios, standardized mean difference and weighted mean difference. GRADE, grading of recommendations, assessment, development and evaluation; HR, hazard ratio; OR, odds ratio; RR, risk ratio; SMD, standardized mean difference; WMD, weighted mean difference; 95% Cl, 95% confidence intervals.

Evidence from nine meta-analyses encompassing 14 pooled estimates substantiated a consistent link between tooth loss and increased risk across various cardiovascular conditions (Figures 2–4) (13, 22–29). Specifically, tooth loss was significantly linked to a higher incidence of cardiovascular diseases (CVDs) (HR = 1.02, 95% CI: 1.01–1.03) (13), coronary heart disease (CHD) (RR = 1.34, 95% CI: 1.10–1.63) (25), stroke (RR = 1.39, 95% CI: 1.13–1.65) (30), and hypertension (OR = 1.20, 95% CI: 1.10–1.30) (credibility assessment class II to IV; GRADE assessment: very low) (28).

Furthermore, a recent dose–response meta-analysis reinforced these findings by revealing that each incremental loss of two teeth was associated with a 3% increase in the risk of both CHD (class III; very low) and stroke (class III; moderate) (23). Two studies reported that tooth loss was associated with an increased risk of mortality related to CVD (HR = 1.66, 95% CI: 1.32–2.09; class III; very low) and all-cause mortality (HR = 1.94, 95% CI: 1.19–3.15; class IV; very low) (13). A dose–response analysis further revealed that each additional 10-tooth loss was associated with a 15% increase in all-cause mortality; this risk increased with increasing tooth loss, with a 33% increase for every additional 20 teeth lost and a 57% increase for the loss of all 32 teeth (class II; moderate) (26). Conversely, three meta-analyses indicated that patients with hypertension (class II; very low) (28), stroke (class III; low) (24), or peripheral artery disease (class IV; very low) (29) had significantly more tooth loss, supporting a bidirectional relationship between oral and cardiovascular health.

### Synthesis of evidence linking tooth loss to cancer risk

The association between tooth loss and cancer risk was evaluated in seven meta-analyses that collectively reported eight unique pooled outcomes (Figure 2; Figure 3) (31–37). With respect to esophageal cancer (EC), a pooled analysis of ten case–control studies revealed that increased tooth loss was associated with an increased occurrence of EC (OR = 1.46, 95% CI: 1.27–1.69; class IV; low) (31). Furthermore, a dose–response analysis indicated a 1% increase in the risk of esophageal cancer with each additional tooth lost (class IV; low) (32). Similarly, a meta-analysis indicated that the loss of more than ten teeth was correlated with a 1.59-fold higher risk of lung cancer (RR = 1.59, 95% CI: 1.21–2.11; class III; very low) (37), and a dose–response analysis within the same study revealed a 9% increase in risk with every additional five teeth lost (class III; very low) (37).

Additionally, a dose–response meta-analysis suggested that each incremental loss of two teeth was associated with a 6% increase in the risk of colorectal cancer (CRC) (RR = 1.08, 95% CI: 1.03–1.15; class III; moderate) (33). Moreover, a recent meta-analysis of four case–control studies and four cohort studies revealed a significantly elevated risk of gastric cancer with increasing tooth loss (RR = 1.44, 95% CI: 1.05–1.98; class III; very low) (36). Notably, the evidence linking tooth loss to an increased risk of oral squamous cell carcinoma and pancreatic cancer remains inconclusive and lacks statistical significance (34, 35).

### Synthesis of evidence linking tooth loss to neurodegenerative diseases

Four meta-analyses comprising a total of 11 pooled analyses provided evidence linking tooth loss to an elevated risk of several neurodegenerative diseases (Figures 2–4) (38–41).

### Synthesis of evidence linking tooth loss to cognitive decline

A recent meta-analysis of 18 cohort studies revealed that tooth loss is associated with a 20% increase in the risk of cognitive decline (RR = 1.2, 95% CI: 1.14–1.24; class IV; very low) (40), with this risk further increasing among individuals with fewer than 10 remaining teeth (RR = 1.2, 95% CI: 1–1.44; class IV; very low) and those with edentulism (RR = 1.27, 95% CI: 1.14–1.41; class IV; low) (40). Moreover, a dose–response analysis indicated a 1% increase in the risk of cognitive decline with each additional tooth lost (class III; moderate) (41).

### Synthesis of evidence linking tooth loss to dementia

Individuals with tooth loss have a 1.55 times greater risk of developing dementia (OR = 1.55, 95% CI: 1.41–1.70; class II; very low) (39). A meta-analysis demonstrated that having fewer than 10 remaining teeth was associated with a 22% increase in the risk of dementia (RR = 1.22, 95% CI: 1.11–1.33; class IV; very low) and edentulism (RR = 1.15, 95% CI: 1.1–1.2; class IV; low) (40). Furthermore, a dose–response analysis revealed that each additional tooth lost was associated with a 1% increase in the relative risk of dementia (HR = 1.01, 95% CI: 1.00–1.02; class IV; moderate) (41).

### Synthesis of evidence linking tooth loss to Alzheimer’s disease

Evidence suggests a bidirectional association between tooth loss and Alzheimer’s disease (AD), in which patients with AD have an increased risk of edentulism (OR = 2.26, 95% CI: 1.7–3.01; class III; very low) (38); similarly, tooth loss is associated with a higher risk of AD (RR = 1.12, 95% CI: 1.02–1.23; class IV; very low) (40).

### Synthesis of evidence linking tooth loss to metabolic disorders

Three meta-analyses investigated the relationship between tooth loss and metabolic disorders and reported four pooled outcomes (Figure 2; Figure 4) (42–44). A meta-analysis of 16 cross-sectional studies revealed a bidirectional association between tooth loss and obesity, with obese individuals having higher odds of tooth loss (OR = 1.49) and individuals with tooth loss having higher odds of obesity (OR = 1.41; class IV; very low) (43). Furthermore, compared with individuals without metabolic syndrome (MetS) (class IV; very low), individuals with MetS had significantly fewer teeth (SMD = −2.77) and a greater risk of lacking functional dentition (44). Additionally, a meta-analysis demonstrated that diabetes significantly increased the risk of tooth loss (class III; very low) (42).

### Synthesis of evidence linking tooth loss to mental disorders

Three meta-analyses examined associations with mental health and tooth loss, yielding three pooled estimates (Figure 4) (45–47). The results of two meta-analyses revealed that depression and anxiety were both associated with a significantly increased likelihood of tooth loss, with odds ratios of 1.31 (95% CI: 1.24–1.37; class IV, low) (45) and 1.22 (95% CI: 1.14–1.30; class III, low) (46), respectively. Additionally, individuals with schizophrenia exhibited significantly greater tooth loss (SMD = 0.79, 95% CI: 0.59–0.98; class IV; very low) (47).

### Synthesis of evidence linking tooth loss to respiratory diseases

Patients with chronic obstructive pulmonary disease (COPD) experienced significantly greater tooth loss than individuals without COPD did (MD = −3.73, 95% CI: −5.12 to −2.33; class III; very low) (Figure 2), underscoring the clear association between COPD and impaired oral health (48).

### Results of the critical appraisal of methodological quality

AMSTAR-2 was used to evaluate the methodological quality of the included studies, and detailed results are provided in Supplementary Table S5. All the investigators adopted reasonable literature search methods and considered the risk of bias in the primary studies when analyzing and explaining the findings. Moreover, the authors used appropriate statistical analysis methods for the meta-analyses in all the reports (item 11). However, only four meta-analyses were rated as having high overall confidence on the basis of preestablished systematic review methods prior to this review (item 2) (22, 28, 34, 41). Only 3 reports provided a partial list of excluded studies and their justification (item 7) (13, 42, 45). Nine of the studies included in this analysis provided discussions of bias risk appraisal (item 13), as detailed in Supplementary Table S4. Publication bias was evaluated in 18 of the 27 included reviews, and all these works addressed its potential impact on their research conclusions (item 15).

When evaluated against the noncritical criteria in AMSTAR-2, the overall confidence in the results of all the included meta-analyses was rated as moderate. Importantly, all the meta-analyses received a score of zero because of two key shortcomings: None of the review authors provided a clear rationale for the selection of their study design (AMSTAR item 3), and none reported funding source information for the primary studies included in their analysis (AMSTAR item 10).

## Discussion

This umbrella review provides a comprehensive synthesis of current meta-analytic evidence regarding the association between tooth loss and various adverse health outcomes. Our analysis included 40 unique pooled estimates, including outcomes related to cardiovascular diseases, cancer, neurodegenerative diseases, metabolic disorders, mental health, and respiratory conditions, with no beneficial outcomes identified. According to the pooled analyses, tooth loss, irrespective of the number of teeth involved or the underlying etiology, can constitute a significant clinical indicator of chronic oral disease and potential systemic health impairments. In the pooled statistical analyses, the increased extent of tooth loss was assessed by comparing subgroups with more and fewer missing teeth as well as each additional 1, 2, 5, 10, or 20 or more teeth lost, and this factor was consistently related to an increased risk of negative health consequences (67.5% of the studied outcomes).

On the basis of appraisals following evidence classification criteria, we classified 15% of the synthesized analyses as presenting highly suggestive evidence (class II), which included risk estimates for all-cause mortality, hypertension and dementia. We classified 30% of these analyses as presenting suggestive evidence (class III), including risks of cardiovascular diseases, stroke, mortality related to CVD, gastric cancer, lung cancer, colorectal cancer, cognitive decline, AD, diabetes mellitus, depression, anxiety and chronic obstructive pulmonary disease. Approximately half of the pooled analyses were classified as presenting weak evidence (class IV). The remaining 5% of the synthesized analyses presented nonsignificant evidence, including for oral squamous cell carcinoma and pancreatic cancer. No studies were identified as presenting convincing evidence (class I) in our analysis. Consistent with prior observations, we detected moderate to high heterogeneity in 50% of the combined analyses (Supplementary Table S3).

When the GRADE framework was used, with observational epidemiological research classified as low-quality evidence, the initial evaluation results were retained for approximately 25% of the pooled analyses. These findings indicate that no additional shortcomings were identified on the basis of the GRADE standards and that an additional 10% of the analyses were elevated to a moderate grade because of the presence of a dose–response gradient. Analyses of dose–response trends supported moderate-level evidence for colorectal cancer, cognitive decline, dementia and stroke. The quality of these associations was mainly downgraded because of inconsistent findings or heterogeneity across the original studies as well as insufficient precision in the analyses, as shown by the wide confidence intervals (Supplementary Table S4).

### Potential mechanisms of action

In recent years, many systematic reviews and meta-analyses have explored the link between tooth loss and various health outcomes. However, our findings suggest that these reviews differ significantly in terms of their focus on tooth loss and general health indicators. Tooth loss has been linked to adverse systemic health outcomes via chronic inflammation, microbial dissemination, and functional, nutritional, and psychosocial impairments.

Periodontal disease, which is caused primarily by bacterial infections in the oral cavity, constitutes the main etiology underlying tooth loss and serves as a significant marker of the cumulative inflammatory burden (49). Periodontitis and subsequent tooth loss have been linked to various aging-related diseases. Importantly, persistent challenges from oral pathogens may induce systemic inflammatory responses, which have been implicated in carcinogenesis (50–52). This mechanistic pathway offers a reasonable explanation for the observed associations between tooth loss and cancers of the head and neck, esophagus, stomach, lung, pancreas, and colorectum. Although the evidence for some cancer sites remains inconclusive, the role of chronic inflammation originating from periodontal disease in such cancers provides a biologically credible link connecting tooth loss to an elevated risk of cancer.

As a clinical endpoint of severe oral disease, tooth loss is also associated with a high incidence of CVD (53). The possible mechanisms include poor masticatory function leading to poor diet and elevated cardiovascular risk. Moreover, the systemic spread of oral inflammation and bacteremia can facilitate the progression of atherosclerosis (54). Furthermore, large-scale cohort studies have indicated that periodontitis and tooth loss are associated with increased all-cause mortality, which may be linked to the acceleration of aging-related processes.

In addition, studies have indicated that periodontitis can promote the occurrence of neuroinflammation through systemic inflammation (55). This connection is supported by elevated concentrations of systemic proinflammatory mediators. Systemic inflammation serves as a standalone risk factor for cognitive decline and links other conditions, such as diabetes, hypertension, hypercholesterolemia and aging, to declines in cognitive performance. Tooth loss can also negatively affect cognitive function by decreasing sensorimotor signals from the masticatory system, which is linked to atrophic changes in brain structure. However, impaired masticatory function may play a key role in cognitive decline through reduced chewing capacity, diminished cerebral perfusion, poor nutritional status, and limited social engagement.

Notably, tooth loss is inherently multifactorial and may result from caries, trauma, socioeconomic disparities, limited access to dental care, a prosthetic history, aging, smoking, or other behavioral and systemic determinants. Thus, the extent of tooth loss may serve as an indirect indicator of the chronicity of underlying periodontitis in some cases. If the extent of tooth loss indicates the chronicity of the underlying process (e.g., periodontitis), more chronic conditions (as reflected by complete tooth loss) might be more likely to lead to cognitive decline (56). In future studies, objective measures of masticatory performance could be used to identify independent effects.

The link between periodontitis and neurocognitive decline is hypothesized to involve two nonexclusive pathways. First, periodontitis may promote neuroinflammation via systemic inflammatory signaling. Second, the reduced sensorimotor input linked to chewing loss has been associated with altered brain function and structure. Thus, the extent of tooth loss, which may indicate the chronicity and severity of oral inflammation, may be correlated with the risk of cognitive decline. The lack of evidence may reflect the difficulty of differentiating different forms of cognitive impairment (38, 40). Because patients with AD already show a substantial loss of self-caring ability (including tooth brushing), it may not be surprising that their cognitive dysfunction further exacerbates poor oral hygiene and increases the risk of tooth decay, periodontal diseases, and extensive tooth loss (57). Thus, the extent of tooth loss, which is potentially indicative of the chronicity and severity of oral inflammation, may be correlated with the risk of cognitive deterioration.

Periodontitis and diabetes exhibit a bidirectional and mutually reinforcing relationship (58). Generally, diabetes increases the risk and severity of periodontitis, and the resulting periodontal disease and tooth loss negatively affect glycemic control, which accelerates diabetes-related complications. Moreover, this vicious cycle is often exacerbated by poor oral hygiene habits in individuals with diabetes, such as infrequent brushing and flossing; the consequent progression of periodontitis further undermines metabolic stability and significantly impairs patients’ overall health and quality of life (59).

When tooth loss is regarded as an outcome indicator, various common systemic conditions, such as Alzheimer’s disease, hypertension, diabetes mellitus, obesity, depression, anxiety and stroke, are closely correlated with an increased risk of tooth loss.

In addition to biological mechanisms, tooth loss has independent effects on mental health through psychosocial pathways because the aesthetic and functional impairments associated with tooth loss can diminish self-esteem and hinder social interactions, potentially leading to withdrawal and distress (60). Crucially, individuals with preexisting psychiatric conditions often demonstrate poorer oral hygiene and use dental services less frequently, which further accelerates oral health decline and reinforces a detrimental feedback loop.

In conclusion, the association between tooth loss and systemic health is multifactorial in many cases and includes the pivotal pathways of chronic inflammation and microbial dissemination. Functional and psychosocial mediators are also crucial bidirectional factors. These findings underscore the importance of integrated oral–systemic healthcare strategies and suggest that preventive and therapeutic dental interventions may confer benefits that extend beyond the oral cavity.

### Strengths and limitations

In this umbrella review, we systematically evaluated the breadth and quality of meta-analytic evidence linking tooth loss to various health outcomes. We synthesized comprehensive evidence across diverse domains, including cardiovascular diseases, cancer, neurodegenerative diseases, metabolic disorders, mental health, and respiratory conditions. Moreover, we adhered rigorously to the PRISMA guidelines, employed the AMSTAR-2 tool for methodological quality assessment, utilized the GRADE framework and considered the credibility of the classification criteria. Furthermore, we focused on both categorical and, where available, dose–response associations to provide a more nuanced understanding of the relationships between tooth loss and various health outcomes.

However, some limitations should be acknowledged. First, the findings were derived primarily from observational studies, which provide associative evidence but preclude the establishment of causality because of potential residual confounding by age, sex, ethnicity, smoking status, socioeconomic status, frailty status, nutritional status, educational level, access to dental care, and multimorbidity factors (61). Second, the influence of oral rehabilitation measures (e.g., dentures, implant-supported prostheses) on the observed associations remains largely unexplored, and the potential modifying effects represent an additional source of unmeasured confounding. Moreover, the pooled analyses are limited by significant heterogeneity owing to the inconsistent definition of tooth loss (e.g., edentulism, number of missing teeth, remaining dentition, or functional dentition thresholds) and differences in the enrolled populations, confounding adjustment strategies, geographical contexts, and study designs. Uncertainties were also noted in the assessment of health outcomes and confounder adjustment, further complicating comparisons and affecting the precision and comparability of the results.

### Policy and clinical implications

The findings of this umbrella review suggest that tooth loss may serve as an underappreciated indicator of systemic health burden. Brief questions about oral health and tooth count in assessments of conditions such as cardiovascular disease, diabetes, and cognitive decline should be considered (62). Integrating oral health screening into routine check-ups may offer benefits, but further evaluation is needed to determine the feasibility and impact of such measures. Moreover, educational initiatives targeting both the public and health professionals might help increase awareness of the links between oral and systemic health (57). A collaborative, patient-centered approach is likely to be important for achieving meaningful improvements in health and quality of life among aging populations.

## Conclusion

This umbrella review shows that greater tooth loss is consistently associated with an increased risk of adverse health outcomes. The evidence supports associations between tooth loss and elevated risks of all-cause mortality, cardiovascular disease-related mortality, cardiovascular illnesses, several cancers (esophageal, gastric, lung, and colorectal), metabolic diseases, mental health disorders, and chronic obstructive pulmonary disease. However, evidence linking tooth loss to oral squamous cell carcinoma and pancreatic cancer remains inadequate. Maintaining good oral health is closely linked to general physical well-being, but interventional evidence remains limited. Further research is needed to explore these correlational links and to verify whether oral health-related interventions can help reduce the risk of relevant illnesses.

## Data Availability

The original contributions presented in the study are included in the article/Supplementary material, further inquiries can be directed to the corresponding author/s.

## Glossary

AHRQ: Agency for Healthcare Research and Quality
AMSTAR 2: The Assessment of Multiple Systematic Reviews 2
CAC JBI: Critical Appraisal Checklist recommended by the Joanna Briggs Institute
CIs: confidence intervals
FE: Fixed effect
GRADE: The Grading of Recommendations, Assessment, Development and Evaluation
HR: Hazard Ratio
I2: I-squared
MD: Mean Difference
mDBs: modified Downs and Black scale
MeSH: Medical Subject Headings
MORE: Methodological Evaluation of Observational Research
NA: Not available
NOS: Newcastle-Ottawa Quality scale
OR: Odds Ratio
PRISMA: preferred reporting items for systematic reviews and meta-analyses
RE: Random effects
ROBINS-E Tool: Risk of Bias in Non-Randomized Studies of Exposures
RR: Risk Ratio
SMD: Standardized Mean Difference
USPSTF: US Preventive Services Task Force
WHO: World Health Organization
WMD: Weighted Mean Difference
