# Supplementary material for: Examining the link between tooth loss and multiple health outcomes: a comprehensive synthesis of evidence from meta-analyses
Source: Front Public Health. 2026 Aug 12;14:1857651. doi: 10.3389/fpubh.2026.1857651 (PMC13506681; doi:10.3389/fpubh.2026.1857651)
Supplement: Supplementary file 1 [file Table_1.DOCX]

**Table S1. Umbrella Review: search terms and strategy used**

| Database | Full search string |
| --- | --- |
| PubMed | (“tooth loss”[Mesh] OR “tooth loss”[Title/Abstract] OR “edentulism”[Title/Abstract] OR “teeth loos”[Title/Abstract] OR “tooth lost”[Title/Abstract] OR “teeth lost”[Title/Abstract]  AND  “systematic review”[Title/Abstract] OR “meta-analysis”[Title/Abstract] |
| Web of science | (“tooth loss” OR “teeth loos” OR “tooth lost” OR “teeth lost” OR “edentulism”  AND  (“systematic review” OR “meta-analysis”) |
| MEDLINE | (“tooth loss”[All Fields] OR “teeth loos”[All Fields] OR “tooth lost”[All Fields] OR “teeth lost” [All Fields]OR “edentulism”[All Fields]  AND  (“meta-analysis”[All Fields] OR “metaanaly*”[All Fields] OR “meta regression”[All Fields] OR “metareg*”[All Fields] OR “systematicreview”[All Fields] OR “systematic-review”[All Fields])) |
| Cochrane library | (“tooth loss” OR “tooth loss” OR “edentulism” OR “teeth loos” OR “tooth lost” OR “teeth lost”  AND  “systematic review”[Title/Abstract] OR “meta-analysis”[Title/Abstract] |

**Table S2. The evidence classification criteria**

| **Evidence class** | **Description** |
| --- | --- |
| **Class I: convincing evidence** | >1000 cases (or >20 000 participants for continuous outcomes); statistical significance at *P*<10^−6^ (random effects); no evidence of small study effects and excess significance bias; 95% prediction interval excluded null value; no large heterogeneity (*I*^2^<50%) |
| **Class II: highly suggestive evidence** | >1000 cases (or >20 000 participants for continuous outcomes), statistical significance at *P*<10^−6^ (random effects), and largest study with 95% confidence interval excluding null value |
| **Class III: suggestive evidence** | >1000 cases (or >20 000 participants for continuous outcomes) and statistical significance at *P*<0.001 |
| **Class IV: weak evidence** | Remaining significant associations with *P*<0.05 |
| **NS: non-significant** | *P*>0.05 |

**Table S3. Umbrella review meta-analysis of tooth loos ans health outcomes**

| **Systemic condition/marker** | **tooth lose as Exposure (E) or Outcome (O)** | **Author and year of publication** | **measurement of Tooth loss** | **No. of cases/total** | **meta-analysis metric** | **Estimates(95%CI)** | **Effects model** | **I^2^ (%)** | **Meta,P** | **Credibility** |
| --- | --- | --- | --- | --- | --- | --- | --- | --- | --- | --- |
| **Cardiovascular Diseases** | | | | | | | | | | |
| **cardiovascular diseases related events** | E | Beukers et al.(2021) | 0 teeth remaining vs. 1-32 teeth (ref.) | 4,697,021 | HR | 2.92 (1.77, 4.82) | random effects | 99% | p＜0.05 | IV:weak |
| **cardiovascular diseases** | E | Cheng et al.(2018) Dose-Response | increasing per 2 of tooth loss | 879084 | RR | 1.03 (1.02, 1.04) | random effects | 88.30% | p < 0.001 | III:suggestive |
| **Coronary heart disease** | E | Humphrey et al. (2008) | 0-10 tooth missing | 112273 | RR | 1.34 (1.10, 1.63) | random effects | NA | p=0.02 | IV:weak |
| **hypertension** | E | Xu et al. (2022) | severe tooth loss | 1,224,821 | OR | 1.20 (1.10, 1.30) | random effects | 40.02% | p=0.00 | II:highly suggetstive |
| **hypertension** | O | Xu et al.(2022) | hypertension as exposure | 1,224,821 | OR | 1.35 (1.07, 1.62) | random effects | 51.10% | p=0.00 | II:highly suggetstive |
| **peripheral artery disease** | O | Yang et al.(2018) | the number of teeth missing | 4307 | WMD | 3.75 (1.31;6.19) | random effects | 56.90% | p＜0.05 | IV:weak |
| **stroke** | O | Dai et al.(2015) | the number of teeth present | 1191 | SMD | -0.33(-0.44;-0.21）) | fixed effects | 39.80% | p < 0.0001 | III:suggestive |
| **stroke** | E | Lafon et al. (2014) | more tooth loss | ranged from 1137 to 51 529 | HR; RR | 1.39 (1.13,1.65) | random effects | NA | NA | IV:weak |
| **stroke** | E | Cheng et al.(2018) Dose-Response | increasing per 2 of tooth loss | 879084 | RR | 1.03 (1.03, 1.04) | fixed effects | 46.70% | p < 0.001 | III:suggestive |
| **CVD mortality** | E | Aminoshariae et al.(2024) | edentulous or less than 10 teeth present | 90305 | HR | 1.66(1.32, 2.09) | random effects | 82.42% | p=.0000 | III:suggestive |
| **All-cause mortality** | E | Beukers et al.(2021) | 0 teeth vs. 1-32 teeth (ref.) | 4,711,667 | HR | 1.94 (1.19, 3.15) | Random effects | 100% | p＜0.05 | IV:weak |
| **All-cause mortality** | E | Peng et al.(2019) Dose-Response | each 10-tooth loss | 306807 | RR | 1.15 (1.11,1.19) | random effects | 80.10% | p=0.000 | II:highly suggetstive |
| **All-cause mortality** | E | Peng et al.(2019) Dose-Response | each 20-tooth loss | 306807 | RR | 1.33(1.23,1.42) | random effects | 79.90% | p=0.000 | II:highly suggetstive |
| **All-cause mortality** | E | Peng et al.(2019) Dose-Response | each 32-tooth loss | 306807 | RR | 1.57(1.41,1.75) | random effects | 79% | p=0.000 | II:highly suggetstive |
| **Cancer** | | | | | | | | | | |
| **Head and Neck Cancer** | E | Mahuli et al.(2023) | lost＞5 teeth,lost＞16 teeth,lost＞20 teeth,only five present | 20513 | OR | 1.13 (0.99,1.26) | random effects | 71.70% | p＜0.001 | NS |
| **Esophageal Cancer** | E | Chen et al. (2015) | more teeth loss:Any lost teeth,16–32 lost teeth,24–32 lost  teeth,Edentulous | 107028 | OR | 1.46 (1.27, 1.69) | fixed effects | 29.90% | p＜0.05 | IV:weak |
| **Esophageal Cancer** | E | Chen et al.(2016) Dose-Response | every 1 tooth loss | 88274 | RR | 1.01 (1.00, 1.03) | fixed effects | 13.50% | p＜0.05 | IV:weak |
| **Gastric Cancer** | E | Yin et al.(2016) | NA | 245752848 | RR | 1.44 (1.05, 1.98) | random effects | 71.30% | p=0.001 | III:suggestive |
| **lung cancer** | E | Yoon et al.(2022) | lost＞10 teeth | 62821 | HR | 1.59 (1.21, 2.11) | fixed effects | NA | p＜0.001 | III:suggestive |
| **lung cancer** |  | Yoon et al.(2022) Dose-Response | every five teeth lost | 252178 | HR | 1.09(1.07,1.11) | random effects | NA | p＜0.001 | IV:weak |
| **pancreatic cancer** | E | Xu (2022) | more tooth loss | 1352256 | HR | 1.19 (0.97, 1.46) | random effects | NA | p＞0.05 | NS |
| **colorectal cancer** | E | Ma et al. (2018) Dose-Response | every two tooth loss | 160182 | RR | 1.06(1.02, 1.11) | fixed effects | 20.50% | p＜0.001 | III:suggestive |
| **Neurodegenerative diseases** | | | | | | | | | | |
| **Cognitive Decline** | E | Qi et al.(2021) Dose-Response | each addional tooth loss | 38763 | HR | 1.01(1.01,1.02) | random effects | I² > 50% | p＜0.05 | III:suggestive |
| **Cognitive Decline** | E | Li et al. (2023) | more tooth loss | 356297 | RR | 1.2 (1.14,1.26) | random effects | 42.30% | p=0.04 | IV:weak |
| **Cognitive Decline** | E | Li et al. (2023) | remaining teeth ＜10 | 356297 | RR | 1.2(1,1.44) | random effects | 78.40% | p＜0.05 | IV:weak |
| **Cognitive Decline** | E | Li et al.(2023) | edentulism | 356297 | RR | 1.27(1.14,1.41) | fixed effects | 16.20% | p＜0.05 | IV:weak |
| **Dementia** | E | Fang et al.（2018) | the number of teeth missing and present | 561846 | OR | 1.55 (1.41,1.70) | fixed effects | 28.00% | p＜0.001 | II:highly suggetstive |
| **Dementia** | E | Li et al. (2023) | remaining teeth ＜10 | 356297 | RR | 1.22(1.11,1.33) | random effects | 63.70% | p＜0.05 | IV:weak |
| **Dementia** | E | Li et al. (2023) | edentulism | 356297 | RR | 1.15(1.1,1.2) | fixed effects | 37.80% | p＜0.05 | IV:weak |
| **Dementia** | E | Qi et al.(2021) Dose-Response | each addional tooth loss | 38763 | HR | 1.01(1.00,1.02) | random effects | 48.10% | p=0.037 | IV:weak |
| **Alzheimer's Disease** | O | Dioguardi et al.(2020) | edentulism | 13472 | OR | 2.26(1.7,3.01) | random effects | 75% | p < 0.00001 | III:suggestive |
| **Alzheimer's Disease** | E | Li et al.(2023) | more tooth loss | NA | RR | 1.12 (1.02,1.23) | random effects | 63.90% | p=0.002 | IV:weak |
| **Metabolic Disorders** | | | | | | | | | | |
| **diabetes mellitus** | O | Ahmadinia et al.(2022) | risk of teeth loss | 677532 | OR | 1.87 (1.62, 2.13) | random effects | 95.90% | p＜0.001 | III:suggestive |
| **Metabolic Syndrome** | O | Souza et al.(2019) | number of teeth | 26750 | SMD | -2.77 (-4.56,  -0.98) | random effects | 100% | p=0.02 | IV:weak |
| **Obesity** | O | Nascimento et al. (2013) | risk of teeth loss | 7994 | OR | 1.49 (1.20, 1.86) | random effects | 83.50% | p＜0.05 | IV:weak |
| **Obesity** | E | Nascimento et al. (2013) | any tooth loss | 3841 | OR | 1.41 (1.11, 1.79) | random effects | 77.70% | p＜0.05 | IV:weak |
| **Mental disorders** | | | | | | | | | | |
| **Schizophrenia** | O | Sun et al.(2021) | missing teeth | 712 | SMD | 0.79 (0.59, 0.98) | fixed effects | 19 | p＜0.00001 | IV:weak |
| **Depression** | O | Cademartori et al. (2018) | more tooth loss | 1553 to 5419019 | OR | 1.31 (1.24, 1.37) | random effects | 13.40% | p＜0.05 | IV:weak |
| **depression and anxiety** | O | Kisely et al.(2016) | more tooth loss | 334,503 | OR | 1.22 (1.14, 1.30) | fixed effects | 23.20% | p＜0.0004 | III:suggestive |
| **Respiratory diseases** | | | | | | | | | | |
| **Chronic Obstructive Pulmonary Disease** | O | Shi et al. (2018) | remaining teeth | 23960 | WMD | -3.73 (-5.12,-2,33) | Random effects | 94.50% | p＜0.001 | III:suggestive |

Note: HR=Hazard Ratio; MD=Mean Difference; OR=Odds Ratio; RR=Risk Ratio; SMD=Standardized Mean Difference; WMD=Weighted Mean Difference

**Table S4. Quality Assessment Using the GRADE Framework of Each Pooled Analysis Assessing Associations Between tooth loss and Health Outcomes**

| **Systemic condition/marker** | **tooth lose as Exposure (E) or Outcome (O)** | **Study** | **Studies，n** | **Risk of bias** | **Inconsistency I2>50 /Serious** | **Indirectness** | **Imprecision** | **other considerations** | **GRADE** |
| --- | --- | --- | --- | --- | --- | --- | --- | --- | --- |
| **Cardiovascular Diseases** | | | | | | | | | |
| **cardiovascular diseases related events** | E | Beukers et al.(2021) | 44 | Not Serious | Serious^a^ | Not Serious | Serious^b^ | None | ⨁◯ ◯ ◯ Very low |
| **cardiovascular diseases** | E | Cheng et al.(2018) Dose-Response | 17 | Not Serious | Serious^a^ | Not Serious | Not Serious | Dose-Response | ⨁⨁◯ ◯ low |
| **Coronary heart disease** | E | Humphrey et al. (2008) | 7 | Not Serious | Serious^a^ | Not Serious | Not Serious | None | ⨁◯ ◯ ◯ Very low |
| **hypertension** | E | Xu et al. (2022) | 28 | Not Serious | Not Serious | Not Serious | Not Serious | None | ⨁⨁◯ ◯ low |
| **hypertension** | O | Xu et al.(2022) | 56 | Not Serious | Serious^a^ | Not Serious | Serious^b^ | None | ⨁◯ ◯ ◯ Very low |
| **peripheral artery disease** | O | Yang et al.(2018) | 7 | Not Serious | Serious^a^ | Not Serious | Not Serious | None | ⨁◯ ◯ ◯ Very low |
| **stroke** | O | Dai et al.(2015) | NA | Not Serious | Not Serious | Not Serious | Not Serious | None | ⨁⨁◯ ◯ low |
| **stroke** | E | Lafon et al.(2014) | 9 | Not Serious | Serious^a^ | Not Serious | Not Serious | None | ⨁◯ ◯ ◯ Very low |
| **stroke** | E | Cheng et al.(2018) Dose-Response | 17 | Not Serious | Not Serious | Not Serious | Not Serious | Dose-Response | ⨁⨁⨁◯ Moderate |
| **CVD mortality** | E | Aminoshariae et al.(2024) | 12 | Not Serious | Serious^a^ | Not Serious | Not Serious | None | ⨁◯ ◯ ◯ Very low |
| **All-cause mortality** | E | Beukers et al.(2021) | 14 | Not Serious | Serious^a^ | Not Serious | Serious^b^ | None | ⨁◯ ◯ ◯ Very low |
| **All-cause mortality** | E | Peng et al.(2019) Dose-Response | 17 | Not Serious | Serious^a^ | Not Serious | Not Serious | Dose-Response | ⨁⨁◯ ◯ low |
| **All-cause mortality** | E | Peng et al.(2019) Dose-Response | 17 | Not Serious | Serious^a^ | Not Serious | Not Serious | Dose-Response | ⨁⨁◯ ◯ low |
| **All-cause mortality** | E | Peng et al.(2019) Dose-Response | 17 | Not Serious | Serious^a^ | Not Serious | Not Serious | Dose-Response | ⨁⨁◯ ◯ low |
| **Cancer** | | | | | | | | | |
| **Head and Neck Cancer** | E | Mahuli et al.(2023) | 27 | Not Serious | Serious^a^ | Not Serious | Serious^b^ | None | ⨁◯ ◯ ◯ Very low |
| **Esophageal Cancer** | E | Chen et al. (2015) | 10 | Not Serious | Not Serious | Not Serious | Not Serious | None | ⨁⨁◯ ◯ low |
| **Esophageal Cancer** | E | Chen et al.(2016) Dose-Response | 10 | Not Serious | Serious^a^ | Not Serious | Not Serious | Dose-Response | ⨁⨁◯ ◯ low |
| **Gastric Cancer** | E | Yin et al.(2016) | 9 | Not Serious | Serious^a^ | Not Serious | Serious^b^ | None | ⨁◯ ◯ ◯ Very low |
| **lung cancer** | E | Yoon et al.(2022) | 6 | Not Serious | Serious^a^ | Not Serious | Serious^b^ | None | ⨁◯ ◯ ◯ Very low |
| **lung cancer** | E | Yoon et al.(2022) Dose-Response | 8 | Not Serious | Serious^a^ | Not Serious | Not Serious | None | ⨁⨁◯ ◯ low |
| **pancreatic cancer** | E | Xu (2022) | 17 | Not Serious | Serious^a^ | Not Serious | Serious^b^ | None | ⨁◯ ◯ ◯ Very low |
| **colorectal cancer** | E | Ma et al. (2018) Dose-Response | 6 | Not Serious | Not Serious | Not Serious | Not Serious | Dose-Response | ⨁⨁⨁◯ Moderate |
| **Neurodegenerative diseases** | | | | | | | | | |
| **Cognitive Decline** | E | Qi et al.(2021) Dose-Response | 3 | Not Serious | Serious^a^ | Not Serious | Not Serious | Dose-Response | ⨁⨁⨁◯ Moderate |
| **Cognitive Decline** | E | Li et al. (2023) | 8 | Not Serious | Not Serious | Not Serious | Not Serious | None | ⨁⨁◯ ◯ low |
| **Cognitive Decline** | E | Li et al. (2023) | 5 | Not Serious | Serious^a^ | Not Serious | Not Serious | None | ⨁◯ ◯ ◯ Very low |
| **Cognitive Decline** | E | Li et al.(2023) | 3 | Not Serious | Not Serious | Not Serious | Not Serious | None | ⨁⨁◯ ◯ low |
| **Dementia** | E | Fang et al.（2018) | 21 | Not Serious | Serious^a^ | Not Serious | Not Serious | None | ⨁◯ ◯ ◯ Very low |
| **Dementia** | E | Li et al. (2023) | 9 | Not Serious | Serious^a^ | Not Serious | Not Serious | None | ⨁◯ ◯ ◯ Very low |
| **Dementia** | E | Li et al. (2023) | 6 | Not Serious | Not Serious | Not Serious | Not Serious | None | ⨁⨁◯ ◯ low |
| **Dementia** | E | Qi et al.(2021) Dose-Response | 6 | Not Serious | Not Serious | Not Serious | Not Serious | Dose-Response | ⨁⨁⨁◯ Moderate |
| **Alzheimer's Disease** | O | Dioguardi et al.(2020) | 6 | Not Serious | Serious^a^ | Not Serious | Not Serious | None | ⨁◯ ◯ ◯ Very low |
| **Alzheimer's Disease** | E | Li et al.(2023) | 6 | Not Serious | Serious^a^ | Not Serious | Not Serious | None | ⨁◯ ◯ ◯ Very low |
| **Metabolic Disorders** | | | | | | | | | |
| **diabetes mellitus** | O | Ahmadinia et al.(2022) | 22 | Not Serious | Serious^a^ | Not Serious | Not Serious | None | ⨁◯ ◯ ◯ Very low |
| **Metabolic Syndrome** | O | Souza et al.(2019) | 12 | Not Serious | Serious^a^ | Not Serious | Serious^b^ | None | ⨁◯ ◯ ◯ Very low |
| **Obesity** | O | Nascimento et al. (2013) | 3 | Not Serious | Serious^a^ | Not Serious | Not Serious | None | ⨁◯ ◯ ◯ Very low |
| **Obesity** | E | Nascimento et al. (2013) | 2 | Not Serious | Serious^a^ | Not Serious | Not Serious | None | ⨁◯ ◯ ◯ Very low |
| **Mental disorders** | | | | | | | | | |
| **Schizophrenia** | O | Sun et al.(2021) | 3 | Not Serious | Not Serious | Not Serious | Serious^b^ | None | ⨁◯ ◯ ◯ Very low |
| **Depression** | O | Cademartori et al. (2018) | 16 | Not Serious | Not Serious | Not Serious | Not Serious | None | ⨁⨁◯ ◯ low |
| **depression and anxiety** | O | Kisely et al.(2016) | 26 | Not Serious | Not Serious | Not Serious | Not Serious | None | ⨁⨁◯ ◯ low |
| **Respiratory diseases** | | | | | | | | | |
| **Chronic Obstructive Pulmonary Disease** | O | Shi et al. (2018) | 14 | Not Serious | Serious^a^ | Not Serious | Not Serious | None | ⨁◯ ◯ ◯ Very low |

Note GRADE=Grading of Recommendations, Assessment, Development, and Evaluation

a. *I*^2^ value >50

b. Wide confidence intervals that include null effect and increased risk

**Table S5. The Assessment of Multiple Systematic Reviews – Second Edition (AMSTAR 2) Quality Assessment Tool, by Health Outcome Domains**

| **Authors** | **Outcomes investigated** | **Year** | **1*** | **2*** | **3*** | **4*** | **5*** | **6*** | **7*** | **8*** | **9*** | **10*** | **11*** | **12*** | **13*** | **14*** | **15*** | **16*** |
| --- | --- | --- | --- | --- | --- | --- | --- | --- | --- | --- | --- | --- | --- | --- | --- | --- | --- | --- |
| **Ahmadinia et al.** | **Metabolic Disorders** | **2022** | Yes | No | No | Partial Yes | Yes | No | Partial Yes | Yes | Partial Yes | No | Yes | Yes | Yes | Yes | Yes | Yes |
| **Aminoshariae et al.** | **Cardiovascular Diseases** | **2024** | Yes | Partial Yes | No | Partial Yes | Yes | Yes | No | Yes | Partial Yes | No | Yes | Yes | Yes | Yes | Yes | Yes |
| **Beukers et al.** | **Cardiovascular Diseases** | **2021** | Yes | No | No | Partial Yes | Yes | Yes | Partial Yes | Yes | Partial Yes | No | Yes | Yes | No | Yes | No | Yes |
| **Cademartori et al.** | **Mental disorders** | **2018** | Yes | No | No | Partial Yes | Yes | Yes | Partial Yes | Partial Yes | Partial Yes | No | Yes | No | No | Yes | No | Yes |
| **Chen et al.** | **Cancer** | **2015** | Yes | No | No | Partial Yes | Yes | Yes | No | Partial Yes | Partial Yes | No | Yes | No | No | Yes | Yes | Yes |
| **Chen et al.** | **Cancer** | **2016** | Yes | No | No | Partial Yes | Yes | Yes | No | Partial Yes | Partial Yes | No | Yes | No | No | Yes | Yes | Yes |
| **Cheng et al.** | **Cardiovascular Diseases** | **2018** | Yes | No | No | Partial Yes | Yes | Yes | No | Yes | Partial Yes | No | Yes | Yes | Yes | Yes | Yes | Yes |
| **Dai et al.** | **Cardiovascular Diseases** | **2015** | Yes | No | No | Partial Yes | Yes | Yes | No | Partial Yes | Partial Yes | No | Yes | No | No | Yes | No | Yes |
| **Dioguardi et al.** | **Neurodegenerative diseases** | **2020** | Yes | No | No | Partial Yes | No | Yes | No | No | Partial Yes | No | Yes | Yes | No | Yes | No | Yes |
| **Fang et al.** | **Neurodegenerative diseases** | **2018** | Yes | No | No | Partial Yes | No | Yes | No | Yes | Partial Yes | No | Yes | Yes | Yes | Yes | Yes | Yes |
| **Humphrey et al.** | **Cardiovascular Diseases** | **2008** | Yes | No | No | Partial Yes | Yes | Yes | No | Partial Yes | Partial Yes | No | Yes | Yes | No | Yes | No | Yes |
| **Kisely et al.** | **Mental disorders** | **2016** | No | No | No | Partial Yes | Yes | Yes | No | Yes | Partial Yes | No | Yes | No | No | Yes | Yes | Yes |
| **Lafon et al.** | **Cardiovascular Diseases** | **2014** | Yes | No | No | Partial Yes | Yes | No | No | Partial Yes | Partial Yes | No | Yes | No | Yes | Yes | No | Yes |
| **Li et al.** | **Neurodegenerative diseases** | **2023** | Yes | No | No | Partial Yes | No | Yes | No | Yes | Partial Yes | No | Yes | Yes | Yes | Yes | Yes | Yes |
| **Ma et al.** | **Cancer** | **2018** | Yes | No | No | Partial Yes | No | No | No | Yes | Partial Yes | No | Yes | No | No | Yes | Yes | Yes |
| **Mahuli et al.** | **Cancer** | **2023** | Yes | Partial Yes | No | Partial Yes | Yes | Yes | No | Partial Yes | Partial Yes | No | Yes | Yes | Yes | Yes | Yes | Yes |
| **Nascimento et al.** | **Metabolic Disorders** | **2016** | Yes | No | No | Partial Yes | Yes | Yes | No | Partial Yes | Partial Yes | No | Yes | Yes | No | Yes | Yes | Yes |
| **Peng et al.** | **Cardiovascular Diseases** | **2019** | Yes | No | No | Partial Yes | Yes | Yes | No | Partial Yes | Partial Yes | No | Yes | No | No | Yes | Yes | Yes |
| **Qi et al.** | **Neurodegenerative diseases** | **2021** | Yes | Partial Yes | No | Partial Yes | Yes | Yes | No | No | Partial Yes | No | Yes | No | Yes | Yes | Yes | Yes |
| **Shi et al.** | **Respiratory diseases** | **2018** | Yes | No | No | Partial Yes | Yes | Yes | No | Yes | Partial Yes | No | Yes | Yes | Yes | Yes | Yes | Yes |
| **Souza et al.** | **Metabolic Disorders** | **2019** | Yes | No | No | Partial Yes | Yes | Yes | No | Yes | Partial Yes | No | Yes | Yes | No | Yes | No | Yes |
| **Sun et al.** | **Mental disorders** | **2021** | Yes | No | No | Partial Yes | Yes | Yes | No | Yes | Partial Yes | No | Yes | No | No | Yes | No | Yes |
| **Xu et al.** | **Cardiovascular Diseases** | **2022** | Yes | Partial Yes | No | Partial Yes | Yes | Yes | No | No | Partial Yes | No | Yes | Yes | No | Yes | Yes | Yes |
| **Xu et al.** | **Cancer** | **2022** | Yes | No | No | Partial Yes | Yes | Yes | No | Yes | Partial Yes | No | Yes | No | No | Yes | Yes | Yes |
| **Yang et al.** | **Cardiovascular Diseases** | **2018** | Yes | No | No | Partial Yes | Yes | Yes | No | Yes | Partial Yes | No | Yes | No | No | Yes | No | Yes |
| **Yin et al.** | **Cancer** | **2016** | Yes | No | No | Partial Yes | Yes | Yes | No | Yes | Partial Yes | No | Yes | No | No | Yes | Yes | Yes |
| **Yoon et al.** | **Cancer** | **2022** | Yes | No | No | Partial Yes | Yes | Yes | No | Partial Yes | Partial Yes | No | Yes | No | No | Yes | Yes | Yes |

| 1* | Did the research questions and inclusion criteria for the review include the components of PICO? |
| --- | --- |
| **2*** | **Did the report of the review contain an explicit statement that the review methods were established prior to the conduct of the review and did the report justify any significant deviations from the protocol?** |
| 3* | Did the review authors explain their selection of the study designs for inclusion in the review? |
| **4*** | **Did the review authors use a comprehensive literature search strategy?** |
| 5* | Did the review authors perform study selection in duplicate? |
| 6* | Did the review authors perform data extraction in duplicate? |
| **7*** | **Did the review authors provide a list of excluded studies and justify the exclusions?** |
| 8* | Did the review authors describe the included studies in adequate detail? |
| **9*** | **Did the review authors use a satisfactory technique for assessing the risk of bias (RoB) in individual studies that were included in the review?** |
| 10* | Did the review authors report on the sources of funding for the studies included in the review? |
| **11*** | **If meta-analysis was performed, did the review authors use appropriate methods for statistical combination of results?** |
| 12* | If meta-analysis was performed, did the review authors assess the potential impact of RoB in individual studies on the results of the meta-analysis or other evidence synthesis? |
| **13*** | **Did the review authors account for RoB in primary studies when interpreting/discussing the results of the review?** |
| 14* | Did the review authors provide a satisfactory explanation for, and discussion of, any heterogeneity observed in the results of the review? |
| **15*** | **If they performed quantitative synthesis did the review authors carry out an adequate investigation of publication bias (small study bias) and discuss its likely impact on the results of the review?** |
| 16* | Did the review authors report any potential sources of conflict of interest, including any funding they received for conducting the review? |

**Item 2, 4, 7,9,11,13, and 15 of 16 items are considered as critical.**
